# Editorial Notes

**Published:** 1932

**Authors:** 


### Editorial Notes

---

**A Short History of the Bristol General Hospital.**

The Bristol General Hospital celebrates the centenary of its foundation this year. In connection with this event Dr. J. Odery Symes has written a short history of the Charity. From the beginning to the end the book is full of interesting information. The first chapter opens with a quotation from the first report of the Committee which charged itself with the task of founding the new hospital in 1831, the wording of which has a delicious fragrance of the long ago. It refers to "the well-known fact that the Bristol Infirmary has for some time past been incompetent fully to answer the demands made upon the Charity." It is true that the reason given is lack of funds, but we suspect the word "incompetent" was not a wholly accidental choice. As Dr. Symes remarks a little later, "the Infirmary was a stronghold of Toryism." We regret that he has lifted so small a corner of the veil; these bygone controversies and hostilities form piquant reading for posterity. From the outset there was an attempt made to establish a "paying ward" to which lady subscribers might send their maids for a small weekly payment. But this was unsuccessful, only £60 in fees being recovered in eight years. There was also a "paying dispensary" attached, where armed with a subscriber's note a mechanic or labourer might receive advice and medicine for six weeks on payment of 3s. 6d. This plan likewise failed. Dr. Symes names as the real founders George Thomas and Joseph Eaton, two close friends, both of whom were Quakers. The Hospital was formally opened on 1st November, 1832, and it is noteworthy that the Committee regarded the education of medical students as being almost as important a function as the care and relief of the sick in the institution.

In the early days the difficulties of obtaining suitable nurses were those common to all hospitals at that time: but Dr. Symes considers that in 1835 it was fairly efficient, and it is described by contemporary authority as "humane."

Changes in the medical and surgical staff were frequent at first, but in many instances the physicians and surgeons who resigned were re-appointed later. It is regrettable that the Bristol General Hospital possessed no "Richard Smith" to leave on record the inner history and gossip of these comings and goings. Evidently Dr. Fairbrother, who resigned his physicianship in 1853 and was elected in 1856 to the Bristol Royal Infirmary was a man to have met. For his colleagues at the Bristol Royal Infirmary had to write to their Committee asking that his statements should be accepted only on his own personal responsibility. Dr. Symes is sometimes almost too reticent; for instance, he relates that when J. G. Swayne was appointed Physician-Accoucheur it was on condition that he handed over all operations to the surgeons. He concludes, "This rule, however, gradually fell into disuse." Was it really as peaceful a decay as that?

Full justice is done to the influence of men like Symonds and the Fripps, but is a pity that the description of their work, both inside and outside the Hospital, is so compressed that it is difficult for a reader to form any real estimate of their personalities and attainments from this brief history. If one may venture a criticism, we could well spare the accounts of unimportant bazaars and devices for raising funds or descriptions of properties bought and buildings erected in order to know more of the men and women who imparted life to the buildings.

The history of the later years of the Bristol General Hospital is one of almost uninterrupted prosperity: necessary extensions have been financed by many munificent benefactors, public subscriptions have been increased, and at present the Bristol General Hospital may justly court comparison with any general hospital in the country.

**The "Lancet" Commission on Nursing.**

THE Final Report of the *Lancet* Commission on Nursing has now been published. In our issue of Autumn, 1931, we remarked that we should be interested to see how the *Lancet* would propose that hospitals should obtain the nursing staffs they need. Section XIII of the Final Report contains the whole of the recommendations (sixty-one in number) made by the Commission. It is important to observe that these recommendations have the unanimous approval of the Commission. We do not propose to reprint the whole of the recommendations, as the Report can be obtained from the *Lancet* offices for 2s. 9d. post free. They deal with :—

(*a*) *The Gap.*—This means the time between leaving school and attaining the age at which girls can begin nursing. It is recommended that there should be facilities for studying anatomy, physiology and hygiene, so that part of the Preliminary State Examination for Nurses may be passed before entering hospital. A wise and feasible plan.

(*b*) *The Cost of Training ;* (*c*) *Salaries of Trained Nurses ;* (*d*) *Salaries of District Nurses ;* (*e*) *Superannuation.*—Various recommendations are made under these headings, designed to make the nurse's curriculum and career something approaching a livelihood, and less of a life of self-denial ending only too often in penury.

(*f*) *Hours of Duty.*—Many improvements are recommended.

(*g*) *Conditions of Admission and Service.*—It is suggested that the initial contract should be fairer to the probationer than it is at present, whilst the living accommodation and the disciplinary rules should be modernized.

(*h*) *Professional Education.*—Recommendations are made to ensure adequate instruction in recognized training hospitals, as well as to improve the State Examination. There is an irresistible demand put forward that the Final Examination should be confined to nursing treatment, and should not include questions on systematic medicine, surgery, gynæcology or psychiatry. Hitherto the Final State Examination has been adversely criticized on this score.

(*j*) *Recognition of Previous Training.*—It is hoped that some scheme may be evolved for such recognition.

(*k*) *Domestic Work in Hospitals.*—Sufficient ward-maids are recommended to relieve nurses of domestic duties not directly concerned with the patient.

(*l*) *Staffing of Non-Approved Hospitals.*—This recommendation is sufficiently bold an innovation to warrant its being reproduced in full and praised without stint. It is even worth enforcing by special legislation :—"Hospitals which are not approved by the General Nursing Council should not seek to enlist probationers for training, but should be staffed by trained nurses and domestic workers."

(*m*) *Tuberculosis Hospitals.*—It is advised that nurses trained in tuberculosis work should have their names placed in a special section of the "Infectious Diseases" Register ; also that sanatorium work calls for higher pay and longer holidays.

(*n*) *Mental Hospitals.*—Various admirable recommendations are made.

The Report deserves the highest praise and the closest study. Section II, describing the "Evolution of British Nursing," is a scholarly and critical contribution to Social History.

In one paragraph (Section III, 32) dealing with the present position the Report says (we do not know with what degree of justification) that senior nurses ask: "What more do they (*i.e.* prospective probationers) want ?" The main task of the Commission has been to answer this question. The Commissioners have faced the position of a shortage of nurses fairly and squarely, they have weighed the evidence impartially, and they have framed a series of recommendations which the nursing profession should seize upon as their "Charter" and the medical profession should insistently and persistently support.

